# International perspective on military exposure data sources, applications, and opportunities for collaboration

**DOI:** 10.3389/fpubh.2023.1154595

**Published:** 2023-05-05

**Authors:** Amy L. Hall, Trish Batchelor, Laura Bogaert, Robert Buckland, Ali B. Cowieson, Michael Drew, Kate Harrison, David I. McBride, Aaron Schneiderman, Kathryn Taylor

**Affiliations:** ^1^Research Directorate, Veterans Affairs Canada, Charlottetown, PE, Canada; ^2^Department of Veterans’ Affairs, Canberra City, ACT, Australia; ^3^Directorate of Force Health Protection, Department of National Defence, Ottawa, ON, Canada; ^4^Defence Health Directorate, New Zealand Defence Force, Wellington, New Zealand; ^5^Department of Defence, Canberra City, ACT, Australia; ^6^Defence Statistics Health, Bath, United Kingdom; ^7^Department of Preventive and Social Medicine, University of Otago, Dunedin, New Zealand; ^8^Epidemiology Program, US Department of Veterans Affairs, Washington, DC, United States; ^9^Military Performance Division, US Army Research Institute of Environmental Medicine, Natick, MA, United States

**Keywords:** military exposures, epidemiology, veteran health, international collaboration, veteran health care

## Abstract

Military personnel may be exposed to a range of hazards. The assessment, documentation and reporting of military exposure information are important steps to guide health protection, services, and research to support actively serving members and veterans. In 2021, a Working Group of researchers from veteran and defense administrations across the Five Eyes countries (Australia, Canada, New Zealand, the United Kingdom, and the United States) was established to examine large military exposure data sources available in each country, their applications, and opportunities to leverage information across administrations and internationally. We provide a brief summary of this work here to highlight some successful examples of data applications and to elicit interest in this evolving area of exposure science.

## Introduction

Military service personnel may encounter a wide range of hazards through their work. Exposure to various physical (e.g., heat, radiation), chemical (e.g., pollution, solvents), biological (e.g., infectious disease) agents and mentally challenging situations can occur during basic training, regular job duties, or deployment (1). While some hazards may be common to civilian workplaces, others, such as those associated with deployment, are unique to military service and can be challenging to assess (2, 3).

Meanwhile, higher rates of adverse health conditions have been observed in veteran populations relative to their civilian counterparts, including chronic pain and musculoskeletal issues (4, 5), hearing difficulties (4–7), respiratory health conditions (8), and mental health conditions (5, 7, 9, 10).

The potential range and complexity of service-related exposures challenge the connection of such exposures to subsequent health outcomes, particularly when effects arise many years after exposure (11). An ongoing assessment and documentation of military exposures in various contexts, preferably over the course individuals’ careers, can provide important information for research, exposure prevention, and veteran compensation.

### Examination of military exposure data sources across the Five Eyes countries

In 2021, an international Working Group of researchers from veteran and defense administrations across the Five Eyes countries (Australia, Canada, New Zealand, the United Kingdom, and the United States) was established to examine military exposure data sources in each country, their applications, and opportunities to leverage information across administrations and internationally. Findings from this effort are briefly summarized here to highlight examples of current sources and applications, and to elicit interest in this evolving area of exposure science.

A total of 57 military exposure data sources were examined (22 from The United States, 15 from Australia, 10 from The United Kingdom, 8 from Canada, and 2 from New Zealand). “Exposure data” was broadly defined, ranging from hygiene exposure measurements to proxies such as military occupation and deployment history. Across countries, military population surveys and administrative databases were the most common types of data reported; others included hygiene (i.e., exposure measurement) databases, or hybrid (i.e., self-reported information and other). Survey data typically included self-reported exposures by active military members and/or veterans while administrative data included medical records, compensation, and personnel records. Coverage varied with respect to capturing information on currently serving military personnel only, veterans only, or a combination (the latter being most common). The majority of exposure data holders were defense administrations; others included veteran administrations, other government entities, and universities. Identifiable subpopulations included particular operations (e.g., Gulf War, Vietnam), military environments or occupations (e.g., personnel posted on submarines), and service periods (e.g., service within a specified period or released since a certain date).

The predominant purpose of data collection was for research and health promotion efforts. In the US for example, the ongoing Service and Health Among Deployed Veterans (SHADE) study examines the impact of deployment for post 9/11 Gulf War Era Veterans to Central Asia, Southwest Asia and Africa and contains deployment history, occupational exposure history and health outcomes related to pollution exposures (12). Data collection is ongoing and includes surveys, exam data collection, health care records, and temporal meteorological and air quality measures mapped by country of deployment. In Canada, an audiometry database contained in the Canadian Forces Health Evaluations and Reporting Outcomes (CF-HERO) (13) system is used to inform hearing loss prevention efforts by monitoring audiometric measures and self-reported hearing and noise exposure questionnaires of active military members. This database is linkable to the main CF-HERO database by unique service number and is used by internal epidemiologists for research projects and to inform hearing conservation policies, such as the ideal time period between audiometric testing, and identifying and monitoring occupational sub-groups with a higher risk of hearing loss.

In the United Kingdom, The Armed Forces Continuous Attitude Survey (AFCAS) annually examines the views and experiences of active military members while serving, covering various service and operational topics such as deployment frequency and length, pre-operational training, and post-operational support and stress management (14). The findings of the survey are used in research focused on health and well-being of United Kingdom Armed Forces members, as well as to inform training and support policies for military members. Also in the United Kingdom, the Gulf 1 Longitudinal Study on Cancers and Deaths, and the Veteran (2001 onwards) Longitudinal Study on Cancers and Deaths uses administrative database linkages (e.g., payroll data, National Health Service (NHS) General Practitioner registration, death certificates, cancer registrations) to examine military factors related to cancers and deaths in current military members and Veterans. The Gulf 1 study examines the impacts of deployment on health outcomes related to cancers and deaths using individual level data and aggregated to the United Kingdom population data on causes of death (15). The Veteran (2001 onwards) study examines the long term impacts of deployment to Iraq (Operation TELIC) and Afghanistan (Operation HERRICK) on Veterans health outcomes by monitoring causes of death, including suicide and cancer registrations among this cohort (16, 17).

Other purposes of military exposure data collection reported by the Working Group included surveillance (exposure and/or health), service delivery (health focused and other), and workforce management. In Australia, The Defense E-Health System combines dental, medical, mental health, and allied health records to create a single data source used by clinicians to manage health services and provide the anticipated benefits of a single data source to centralize, electronically capture and manage ADF health records, and seamlessly link health data for ADF personnel. (18). The US Defense Manpower Data Center (DMDC) is a central database for the Department of Defense with information on active military members and historical service records for Veterans of the uniformed services (19). It also includes personnel information such as date of birth, rank, sex, component, occupational title, and unit identification as proxy measures of military exposures. It is used to quantify available manpower and collect personnel data to determine military readiness. The DMDC has been used for demographic variables in population health studies, including a study on the health effects from possible exposures to sarin and cyclosarin in Gulf War Veterans where date of birth, gender, marital status, race, branch, rank, and military occupation were obtained through the DMDC (20).

Military exposure data can also be used for benefits claims and compensation purposes (11). Australia’s Department of Veterans Affairs Claims Data was triangulated with findings of a desktop analysis of Australian Defense Force (ADF) initial training courses, a research survey of ADF trainees and staff, and direct observations of training to develop a job exposure matrix through which to understand occupational exposures within training regimes conducted by the ADF as they relate to the development of osteoarthritis of the lower limbs (21).

## Discussion

These examples identified by the Working Group illustrate the value of collecting military exposure information, particularly when links with other data sources can be made. In Canada, the addition of a Veteran identifier to the 2021 Census provides new opportunities for international comparisons (United Kingdom and Australia also added military service census questions in 2021) as well as data linkages across multiple sources. For instance, a cross-departmental initiative involving Statistics Canada, Veterans Affairs Canada, and the Department of National Defense has led to the centralization of military service information obtained from the Census along with historical military records and federal administrative data. This has produced a comprehensive status file of over 2.7 million individuals with Canadian military service over the past 100 years that may be linked to various other health data sources.

A US example is the Individual Longitudinal Exposure Record (ILER), a web application developed by the United States Department of Defense (DoD) with support from the US Department of Veterans Affairs (22). The ILER is designed to create a historic record of individual service related exposures, with soldier identifiers linkable to other administrative data sources or external research projects. Thus, the ILER can be used to identify cohorts and registries based on operations, and to support research examining associations between various military exposures and health outcomes (23). Some examples of current registries include Airborne Hazard and Open Burn Pit, Chemical and Biological Warfare Exposure System, Gulf War Oil Well Fire Smoke, and Depleted Uranium. Clinicians are also able to link DoD exposure data to veterans to support patient care and benefit claims (23). Efforts are underway to develop new linkages with the ILER data, including dosimetry data, deployment health assessments and hearing conservation data, to permit larger research studies and expanded surveillance activities with full operating capability beginning in September 2023 (24). The United Kingdom’s Service Leavers database, an administrative Veteran database with information on service start/end dates and rank at release from service, is linkable to other data sources describing military exposures and health outcomes. This database was applied in a research collaboration between Manchester University and the Ministry of Defense to examine suicide rates, timing between discharge and risk factors for suicide of the United Kingdom Armed Forces Veterans between 1996 and 2005 (25). The Australian Defense Department retains all personnel records of active military members and/or Veterans in their Personnel Management Key Solution (PMKeyS) administrative database, which contains details such as training certificates and qualifications, operation and deployment history, and occupation. This administrative database is currently linked to the National Death Index for ADF members and their suicide reporting system and is being used to identify survey cohorts as well as for population health reporting.

The Working Group also identified challenges to collecting and combining military exposure data with other sources of information. Limited resources to fund studies and hire specialized research staff was a commonly reported issue. National security concerns and strict data protection and governance structures were also identified as roadblocks to linking exposure data with other sources of information. Data collection in active scenarios, such as during international deployments or in combat situations, is understandably not always a top priority, but can result in limited quantification of hazards in these contexts.

Further, the Working Group noted a general lack of sex and gender specific information in exposure data sources across countries. This is an important gap given known differences in occupational exposure patterns and subsequent health risks across men and women (26, 27). The increasing diversity of military populations and expanding scopes of duty in women (2, 28) justifies the need to collect and analyze data to permit differentiation of exposures across women and men, as well as other understudied groups. Such efforts should be prioritized as military and veteran data sources are established and expanded.

There are many other opportunities to develop new resources and leverage existing data. Data scientists and health researchers continue to develop data collection techniques and methods of analyzing human exposures. Data centralization and linkages are key to examining new health questions and traditionally uninvestigated subpopulations with stronger sample sizes. As highlighted previously, there are a number of linkage initiatives underway or planned in multiple countries. While funding is a challenge across research disciplines, there are hopeful examples in the field of military and veteran health, such as special purpose research funding mechanisms in the United States focused on toxic military environmental exposures (29, 30). External collaboration beyond an individual department or country brings additional value in terms of shared data sources and expertise, particularly with respect to standardizing measures and systems in order to facilitate data pooling and comparisons across countries. Such initiatives would ideally feed into the development, co-ordination and review of Defense occupational health systems to reduce adverse health effects in serving personnel and veterans.

## Summary

Our international Working Group has provided a brief summary of the types of military exposure data sources existing across five countries, and examples of their value to inform health care, surveillance, and research in military and veteran populations. It is our hope that this personal view piece will stimulate further discussion on opportunities to leverage existing information and promote ongoing data collection.

## Data availability statement

The datasets presented in this article vary in terms of their public availability. Requests to access the datasets should be directed to amy.hall@veterans.gc.ca.

## Ethics statement

Ethical review and approval was not required for the current study in accordance with the local legislation and institutional requirements. The study did not involve human participants and written informed consent for participation was not required in accordance with the national legislation and the institutional requirements.

## Author contributions

AH and AC drafted this article with intellectual input from all authors. All authors reviewed the article draft, approved the final version for submission, and agree to be accountable for all aspects of the work.

## Funding

This work was conducted through internal funding from Veterans Affairs Canada (Government of Canada).

## Conflict of interest

The authors declare that the research was conducted in the absence of any commercial or financial relationships that could be construed as a potential conflict of interest.

## Publisher’s note

All claims expressed in this article are solely those of the authors and do not necessarily represent those of their affiliated organizations, or those of the publisher, the editors and the reviewers. Any product that may be evaluated in this article, or claim that may be made by its manufacturer, is not guaranteed or endorsed by the publisher.
